# Drugs repurposing in the experimental models of Alzheimer’s disease

**DOI:** 10.1007/s10787-024-01608-7

**Published:** 2025-01-03

**Authors:** Sheer A. Joodi, Weam W. Ibrahim, Mahmoud M. Khattab

**Affiliations:** Department of Pharmacology and Toxicology, Faculty of Pharmacy, Cairo University, ElKasr Elaini Street, Cairo, 11562 Egypt

**Keywords:** Alzheimer’s disease, A*β*42, Nrf2, NF-κ*β*, P38-MAPK, mTOR, And GSK-3*β*

## Abstract

The currently approved drugs for Alzheimer’s disease (AD) are only for symptomatic treatment in the early stages of the disease but they could not halt the neurodegeneration, additionally, the safety profile of the recently developed immunotherapy is a big issue. This review aims to explain the importance of the drugs repurposing technique and strategy to develop therapy for AD. We illustrated the biological alterations in the pathophysiology of AD including the amyloid pathology, the Tau pathology, oxidative stress, mitochondrial dysfunction, neuroinflammation, glutamate-mediated excitotoxicity, insulin signaling impairment, wingless-related integration site/*β*-catenin signaling, and autophagy. Additionally, we demonstrated the different repurposed drugs in the experimental models of AD including the anti-inflammatory, anti-hypertensive, anti-diabetic, antiepileptic, antidepressant and anticancer drugs. Further, we showed the pipeline and FDA approved drugs for AD. The repurposed drugs have a promising therapeutic activity against AD, confirming the value of the drugs repurposing technique to elucidate curative therapy for AD.

**Graphical abstract:**

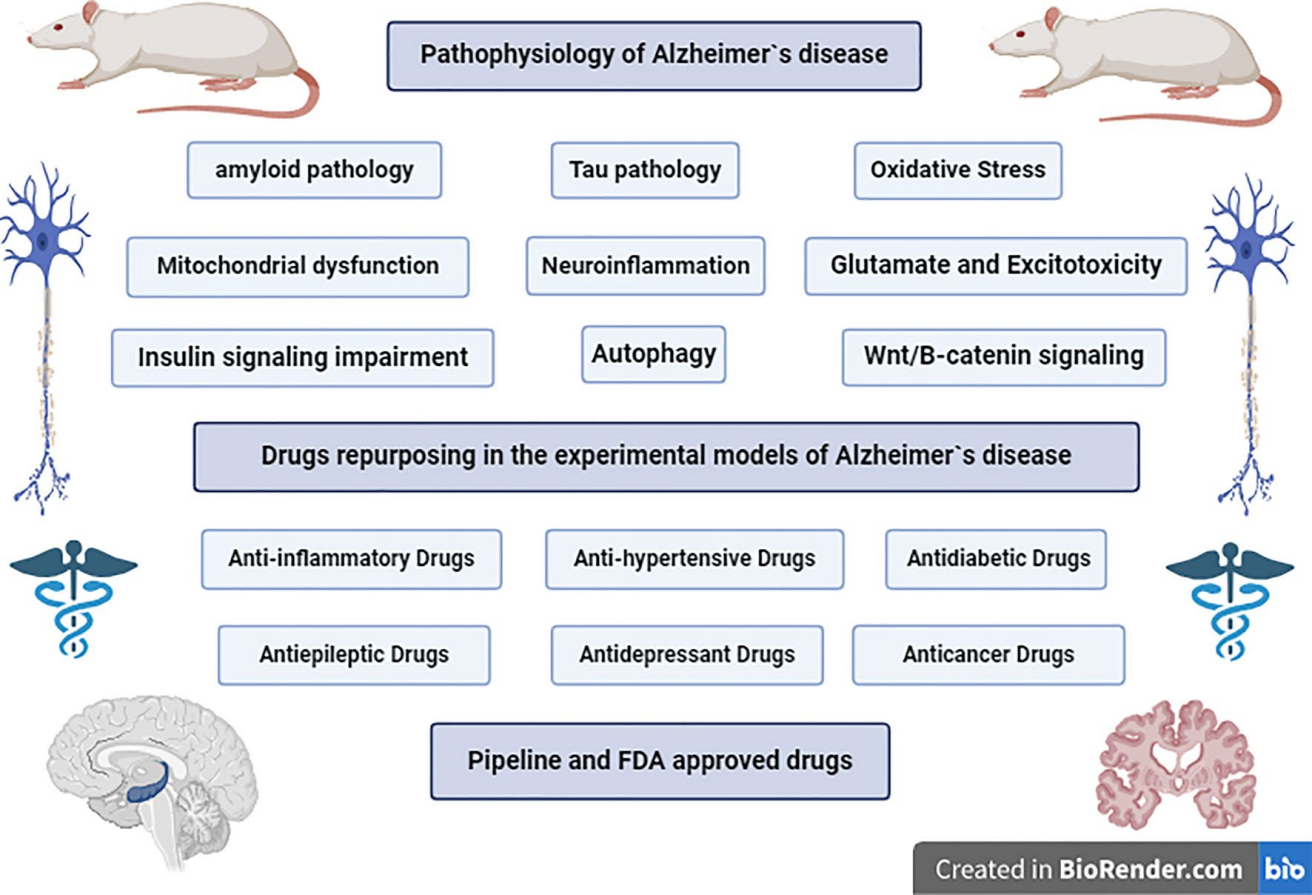

## Introduction

### Pathophysiology of the Alzheimer’s disease (AD)

AD is an age-related neurodegenerative disorder characterized by memory, learning, and decision making impairments. It was discovered by Alois Alzheimer, who analyzed the brain tissue of dead AD patients in 1906. AD is considered as the fifth cause of death in 13% of people over 65, based on the observation of Alzheimer’s association (Folch et al. 2016). Further, the overall predicted prevalence of AD in global population will be quadruple in the next decades, about 114 million patients by 2050 according to the World Health Organization (Langa et al. 2009). Hippocampus, the major brain region of memory processing, is affected by cholinergic transmission. The degeneration of cholinergic neurons in the nucleus basalis of Meynert with consequent loss of cholinergic inputs to the hippocampus and cerebral cortex induce memory impairment. Prior studies recorded declines in choline acetyl-transferase (ChAT) activity, acetylcholine (Ach) release, along with decrements in nicotinic and muscarinic receptors density in the hippocampus and cerebral cortex of postmortem AD brains (Folch et al. 2016). Further, the activity of acetylcholinesterase (AchE) enzyme is enhanced in AD patients, resulting in Ach hydrolysis, decline in Ach level, as well as cholinergic signals impairment with subsequent cognitive decline (Alhazmi and Albratty 2022).

The pathological cascade of AD is initiated by extracellular amyloid-*β*42 (A*β*42) plaques deposition and Tau hyper-phosphorylation, which forms intracellular neurofibrillary tangles (NFTs), leading to oxidative stress, mitochondrial dysfunction, neuroinflammation, as well as, excitotoxicity, and insulin signaling impairment, resulting in neuronal apoptosis (Alhazmi and Albratty 2022). This article will demonstrate the pathophysiology of AD, the repurposed drugs from different classes in the experimental models of AD, pipeline and FDA approved drugs.

#### The amyloid pathology

**Fig. 1:**
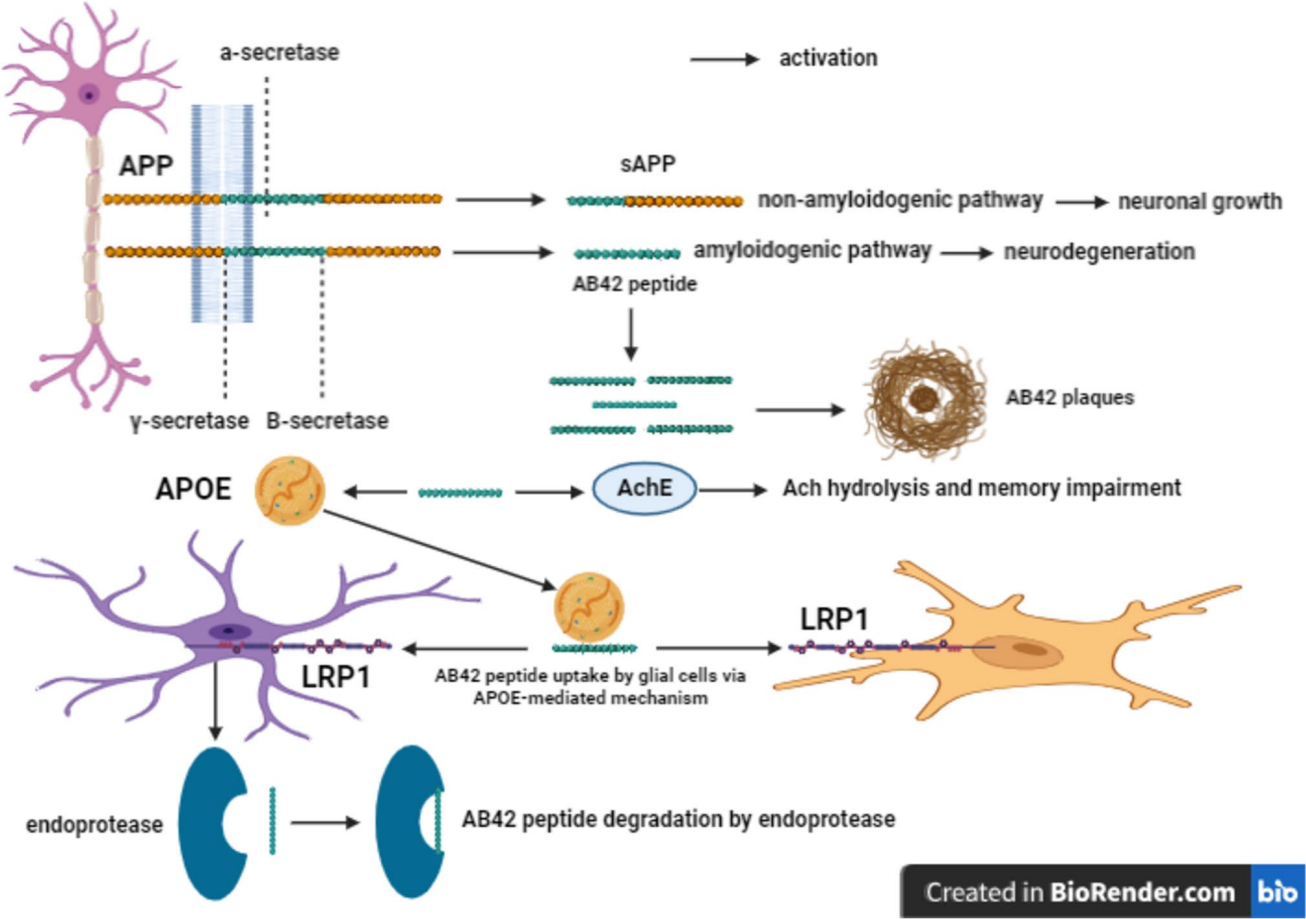
The amyloid pathology. APP amyloid precursor protein, A*β*42 amyloid-*β*42, sAPP soluble amyloid precursor protein, APOE Apolipoprotein E, LRP1 low-density lipoprotein receptor-related protein-1, AchE acetylcholine esterase, Ach acetylcholine

The A*β*42 plaques deposition, the major reason for AD pathology, is induced through the amyloidogenic pathway after the cleavage of amyloid precursor protein (APP), a transmembrane protein in neuronal synapses, by *β*-secretase and *ϒ*-secretase, forming A*β*42 peptides that tend to aggregate and form plaques. Whereas, the non-amyloidogenic pathway occurs following the cleavage of APP by α-secretase, producing soluble APP that mediates neuronal growth and synaptic transmission (Masters et al. 2015; Knopman et al. 2021). A*β*42 peptides enhance AchE activity, resulting in Ach hydrolysis and memory impairment (Teixeira et al. 2019). Additionally, A*β*42 peptides target various neuronal and astrocytic receptors including insulin, frizzled, α7 nicotinic acetylcholine receptor (α7nAChR), *N*-methyl-d-aspartate (NMDA), and APOE (apolipoprotein E) (Armato, Chiarini et al., 2013, Kumar and Singh 2015). A*β*42 peptides are cleared by APOE-mediated mechanism and taken by microglia and astrocytes via low-density lipoprotein receptor-related protein-1, inducing A*β*42 peptides phagocytosis by microglia. Further, A*β*42 peptides are degraded by endoproteases, which are released from astrocytes such as insulin degrading enzyme (IDE), neprilysin (NEP), and matrixmetalloproteinase (MMP) (Masters et al. 2015) **(**Fig. 1**)**.

There are three allelic isoforms of APOE, 7% for APOE2, 78% for APOE3, and 15% for APOE4 in Americans of European descent (Masters et al. 2015). APOE2 reduces AD prevalence as well as APOE3 impedes A*β*42 deposition, on the contrary APOE4 evokes A*β*42 deposition and diminishes the clearance machinery of A*β*42 through competition to bind to A*β*42 receptors on the glial cells (Masters et al. 2015; Long and Holtzman 2019). AD is classified according to the age of onset of symptoms as familial and sporadic AD. In the case of familial AD, represents 2% of diagnosed cases, the cognitive decline begins at 40 years old due to mutation in APP gene (chromosome 21), presenilin-1 (PS1, chromosome 14), and presenilin-2 (PS2, chromosome 1), triggering A*β*42 generation, while sporadic AD is subdivided into early onset if the symptoms begin under 65 years of age (3–5% prevalence) with the rest of the cases represented as late onset (95–97% prevalence) due to e4 allele of the APOE gene (chromosome 19) (Folch, Petrov et al., 2016).

#### The Tau pathology

**Fig. 2:**
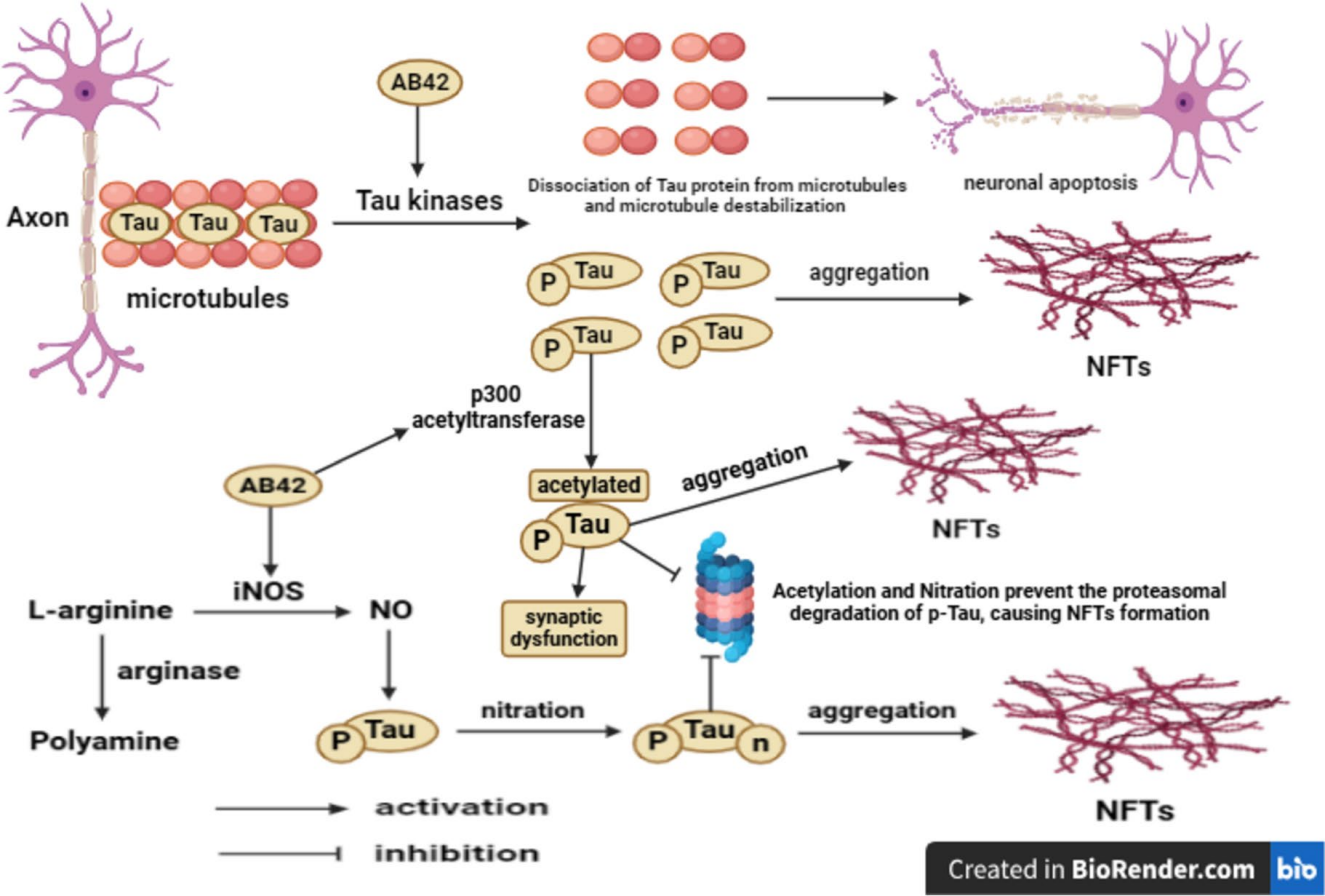
The Tau pathology. A*β*42 amyloid-*β*42, p-Tau phosphorylated Tau, n-Tau nitrated Tau, NFTs neurofibrillary tangles, iNOS inducible nitric oxide synthase, NO nitric oxide

Under the physiological condition, the highly soluble Tau protein promotes microtubule stabilization in neurons especially in axons and facilitates nutrient transport as well as neuronal growth. The binding of Tau protein to microtubules is regulated by phosphorylation process. The imbalance between the catalytic activity of kinases and phosphatases induces Tau hyper-phosphorylation, leading to dissociation of Tau protein from microtubules and microtubule destabilization, mediating neuronal cell death with subsequent formation of insoluble paired helical filament and NFTs (Folch et al. 2016). The formation of A*β*42 plaques provokes Tau hyper-phosphorylation via activating various kinases such as glycogen synthase kinase-3 beta (GSK-3*β*), c-Jun *N*-terminal kinase (JNK), extracellular signal-regulated kinase1/2 (ERK1/2), p38-mitogen-activated protein kinase (P38-MAPK), and mammalian target of rapamycin (mTOR) (Oddo 2012, Folch et al. 2016, Zhang et al. 2021), on the contrary protein phosphatase-2A (PP2A) dephosphorylates Tau protein (Oddo 2012, Folch et al. 2016). Additionally, Tau protein undergoes several post-translational modifications like nitration that impairs the Tau ability to conduct the tubulin assembly (Reynolds et al. 2006; Reyes et al. 2008) and hampers the susceptibility of the phosphorylated Tau (p-Tau) to the ubiquitin proteasome degradation system, causing NFTs formation (Riederer et al. 2009). A*β*42 plaques activate inducible nitric oxide synthase (iNOS) that utilizes l-arginine to produce nitric oxide (NO), resulting in Tau nitration with consequent NFTs formation, while arginase converts l-arginine to polyamine and prevents both NO formation and Tau nitration (Ashour et al. 2021). The acetylation of Tau protein in lysine residues including 174, 274, 280, and 281 reduces the Tau microtubule stabilizing ability, promotes Tau aggregation, and inhibits the proteasomal degradation of p-Tau as well as inducing NFTs formation and synaptic dysfunction (Irwin et al. 2012; Tracy and Gan 2017). It was documented that A*β* peptides induced Tau acetylation through p300 acetyltransferase activation or silent information regulator1 (SIRT1) inhibition in cultured cell lines and transgenic mice models of AD (Tracy and Gan 2017). Further, the acetylated-Tau (lysine 280) showed a distribution pattern similar to p-Tau and it was detected in all stages of the AD especially in moderate to severe cases as reported in postmortem brains of AD patients (Irwin et al. 2012). Furthermore, acetylated-Tau (lysine 174) level increased in the early stage of AD, while high levels of 274 and 281 lysine residues of the acetylated-Tau were recorded in the brains of AD patients in the late stagy of AD (Tracy and Gan 2017). Moreover, salsalate, a prodrug of salicylate, could diminish p300 acetyltransferase activity, acetylated-Tau (lysine 174) level, and Tau-mediated cognitive impairment in PS19 transgenic mice model of frontotemporal dementia (FTD), confirming that the inhibition of Tau acetylation is a promising therapeutic target for Tau pathology in AD (Min et al. 2015) **(**Fig. 2**)**.

#### Oxidative stress and mitochondrial dysfunction

The A*β*42 plaques and NFTs aggravate microglial and astrocytic activation to produce reactive oxygen species (ROS) and inflammatory cytokines, provoking oxidative stress and neuroinflammation (Glass et al. 2010; Feng and Wang 2012). ROS cause oxidative damage to proteins, lipids, and nucleic acid, resulting in AD progression (Butterfield et al. 2001; Di Domenico et al. 2010). Additionally, ROS aggravate neuronal apoptosis through mitochondrial apoptotic pathway featured by increasing pro-apoptotic proteins like Bcl-2-associated x protein (Bax) and decreasing anti-apoptotic proteins like B cell lymphoma-2 (Bcl2) (Rana 2008; Tang et al. 2019; Hao et al. 2020). The accumulation of A*β*42 plaques in mitochondria exacerbates mitochondrial respiratory enzymes complex-II and IV failure, energy depletion, and ROS generation, as well as promoting cytochrome c release which activates caspase-3 with subsequent induction of apoptosis (Kumar and Singh 2015; Noble et al. 2017). There are seven isoforms of nicotinamide adenine dinucleotide phosphate (NADPH) oxidase (NOX) enzyme in neurons, microglia, and astrocytes for ROS generation. The prototypic NOX, NOX2 isoforms, consist of a membrane-bound cytochrome b558 (p22phox and gp91phox subunits) and various regulatory cytosolic subunits (p47phox, p40phox, p67phox, and the GTPase Rac1 or Rac2). The translocation of cytosolic subunits to the membrane and binding to the cytochrome b558 induce NOX activation and superoxide anion generation that is considered as precursor for ROS including hydrogen peroxide (H_2_O_2_), hydroxyl radicals (^•^OH), and peroxynitrite (ONOO^−^) (Ibrahim et al. 2020).

**Fig. 3:**
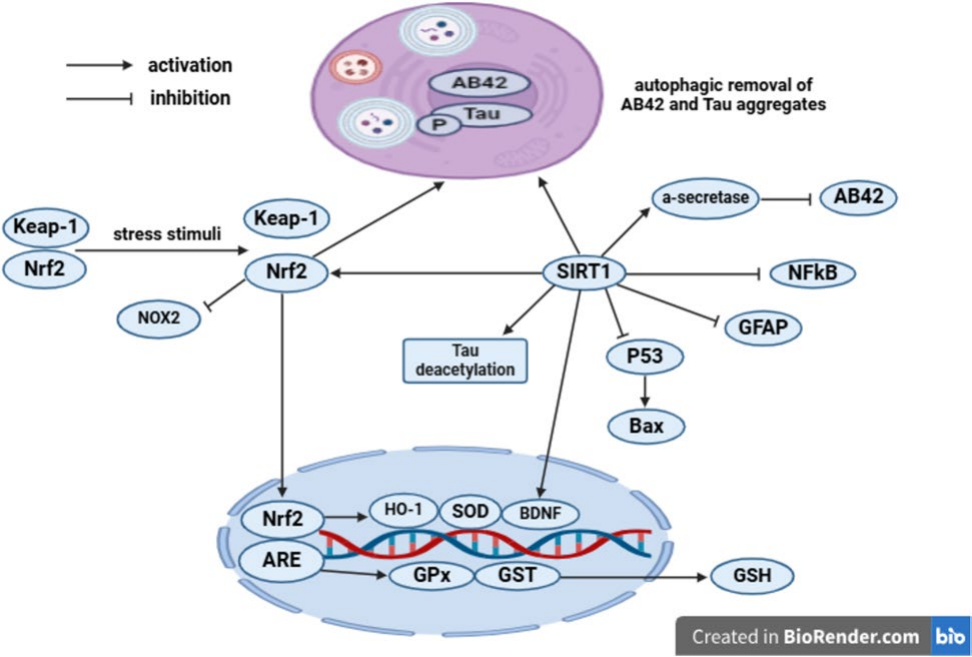
Nrf2 and SIRT1 signaling. A*β*42 amyloid-*β*42, Nrf2 nuclear factor erythroid 2-related factor 2, Keap-1 kelch-like ECH-associated protein-1, ARE antioxidant response elements, HO-1 heme oxygenase-1, SOD superoxide dismutase, GPx glutathione peroxidase, GST glutathione S-transferase, GSH glutathione, NADPH nicotinamide adenine dinucleotide phosphate, NOX NADPH oxidase, NF-κ*β* nuclear factor-kappa *β*, BDNF brain derived neurotrophic factor, SIRT1 silent information regulator1, GFAP glial fibrillary acidic protein, P53 tumor suppressor protein, Bcl-2 B cell lymphoma-2, Bax Bcl-2-associated x protein

The nuclear factor erythroid 2-related factor 2 (Nrf2) is normally located in the cytoplasm and linked to Keap-1 (Kelch-like ECH-associated protein1). Upon stress stimuli, Nrf2 dissociates from Keap-1 and translocates to the nucleus with subsequent binding to antioxidant response elements, resulting in upregulation of detoxifying enzymes such as heme oxygenase-1 (HO-1), superoxide dismutase (SOD), glutathione peroxidase (GPx), and glutathione S-transferase (GST) that produce glutathione (GSH) and free radical scavengers (Vasconcelos et al. 2019, Qu et al. 2020). Further, Nrf2 inhibits NOX2 activity, causing decrement in ROS formation (Ibrahim and Abdel Rasheed 2022). Additionally, Nrf2 facilitates neuronal growth and survival through enhancement of brain derived neurotrophic factor (BDNF) expression (Yao et al. 2021). Moreover, Nrf2 facilitates the autophagic clearance of A*β*42 and p-Tau aggregates in AD (Vasconcelos et al. 2019). SIRT1, a NAD^+^-dependent deacetylase, exerts a neuroprotective activity against oxidative stress, apoptosis, and neuroinflammation, as well as promoting α-secretase-mediated cleavage of the APP (Qin et al. 2006; Ye and Wu 2021). Further, SIRT1 deacetylates Tau protein and facilitates the autophagic clearance of A*β*42 and p-Tau aggregates (Tracy and Gan 2017; Ibrahim et al. 2022). Furthermore, SIRT1 enhances Nrf2 transcriptional activity (Ibrahim and Abdel Rasheed 2022). Additionally, SIRT1 inhibits tumor suppressor protein (P53) and its downstream genes expression like Bax, while facilitating BDNF expression and neuronal survival (Tian et al. 2020; Ibrahim and Abdel Rasheed 2022). Moreover, SIRT1 modulates neuroinflammation by inactivating NF-κ*β* and suppressing astrocytic activity with consequent decline in glial fibrillary acidic protein (GFAP); the marker of astrogliosis (Ibrahim and Abdel Rasheed 2022) (Fig. 3).

#### Neuroinflammation

**Fig. 4:**
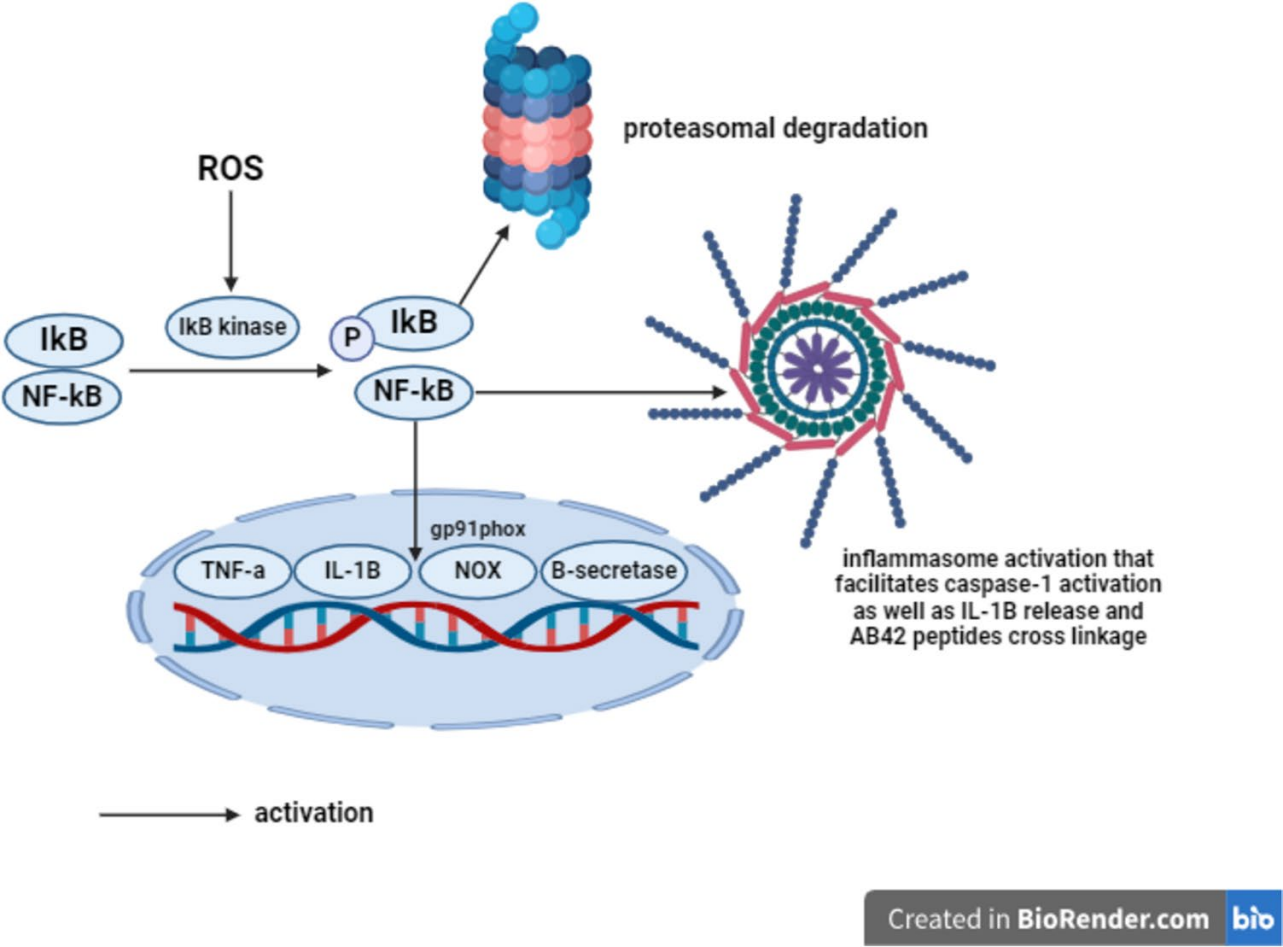
NF-κ*β* signaling. A*β*42 amyloid-*β*42, NF-κ*β* nuclear factor-kappa *β*, IκB inhibition of nuclear factor-kappa *β*, TNF-α tumor necrosis factor-α, IL-1*β* interleukin- 1*β*, NADPH nicotinamide adenine dinucleotide phosphate, NOX NADPH oxidase

Inflammatory cytokines including tumor necrosis factor-α (TNF-α), interleukin- 1*β* (IL-1*β*), and interleukin-6 (IL-6) exacerbate APP expression, A*β*42 generation, neuronal destruction, (Buxbaum et al. 1992; Blasko et al. 2000; Rubio-Perez and Morillas-Ruiz 2012) as well as degeneration of the cholinergic basal forebrain cells and promotion of AchE activity, leading to Ach degradation with subsequent cognitive decline (Akiyama et al. 2000). NF-κ*β* p50/p65 is normally located in the cytoplasm and linked to inhibitor of nuclear factor kappa *β* (Iκ*β*) protein. ROS activate Iκ*β* kinase, facilitating Iκ*β* ubiquitination and p50/p65 dimer translocation to the nucleus (Rawdin et al. 2013; Moustafa et al. 2018). NF-κ*β* promotes the transcription of inflammatory cytokines such as TNF-α and IL-1*β* (Lu et al. 2010). It is also involved in oxidative stress through NOX activation by upregulating gp91phox subunit (Anrather et al. 2006). Additionally, NF-κ*β* enhances the amyloidogenic processing of APP via upregulating *β*-secretase (Tamagno et al. 2012). Moreover, NF-κ*β* inhibits the phagocytic function of microglia to clear A*β*42 peptides (Zhao et al. 2014). Furthermore, NF-κ*β* signaling enhances nucleotide-binding oligomerization domain (NOD)-like receptor protein 3 (NLRP3) inflammasome, resulting in activation of caspase-1-mediated neuronal cell death and secretion of IL-1*β*, additionally, NLRP3 activation is involved A*β*42 plaques formation via promoting A*β*42 peptides cross linkage (Tan et al. 2013; Long and Holtzman 2019) (Fig. 4).

**Fig. 5:**
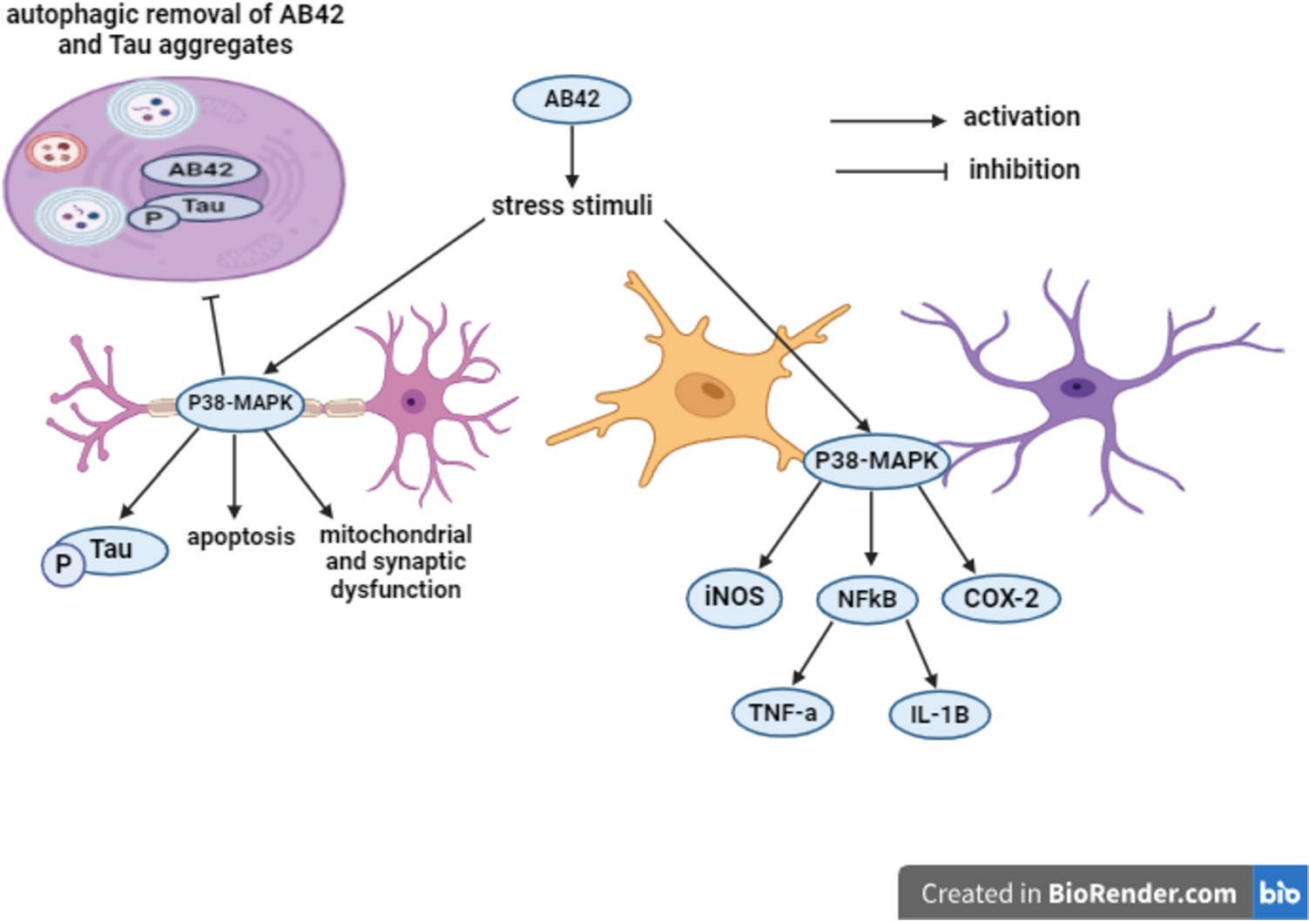
P38-MAPK signaling. A*β*42 amyloid-*β*42, P38-MAPK p38-mitogen-activated protein kinase, p-Tau phosphorylated Tau, NF-κ*β* nuclear factor-kappa *β*, TNF-α tumor necrosis factor-α, IL-1*β* interleukin- 1*β*, COX-2 cyclooxygenase-2, iNOS inducible nitric oxide synthase

Inflammatory cytokines activate Janus Kinase-2/Signal Transducers and Activators of Transcription-3 (JAK2/STAT3) signaling pathway that exacerbates neuroinflammation by enhancing the transcription of inflammatory cytokines like TNF-α (Nishiki et al. 2004; Huang et al. 2008). P38-MAPK, a member of MAPK family, expressed by neurons, microglia, and astrocytes in the brain regions of learning and memory processing, additionally, it is activated by stressful stimuli such as ROS and inflammatory cytokines, accelerating AD pathological cascade. The activation of P38-MAPK in neurons exacerbates Tau hyper-phosphorylation, mitochondrial and synaptic dysfunction, as well as neuronal apoptosis. Further, in response to A*β*42 plaques, P38-MAPK activation in glial cells induces iNOS, cyclooxygenase 2 (COX-2), and NF-κ*β* activation, as well as enhancing TNF-α and IL-1*β* release. Furthermore, P38-MAPK inhibits the autophagic clearance of A*β*42 and p-Tau aggregates (Mansour et al. 2022) **(**Fig. 5**)**.

#### Glutamate and excitotoxicity

**Fig. 6:**
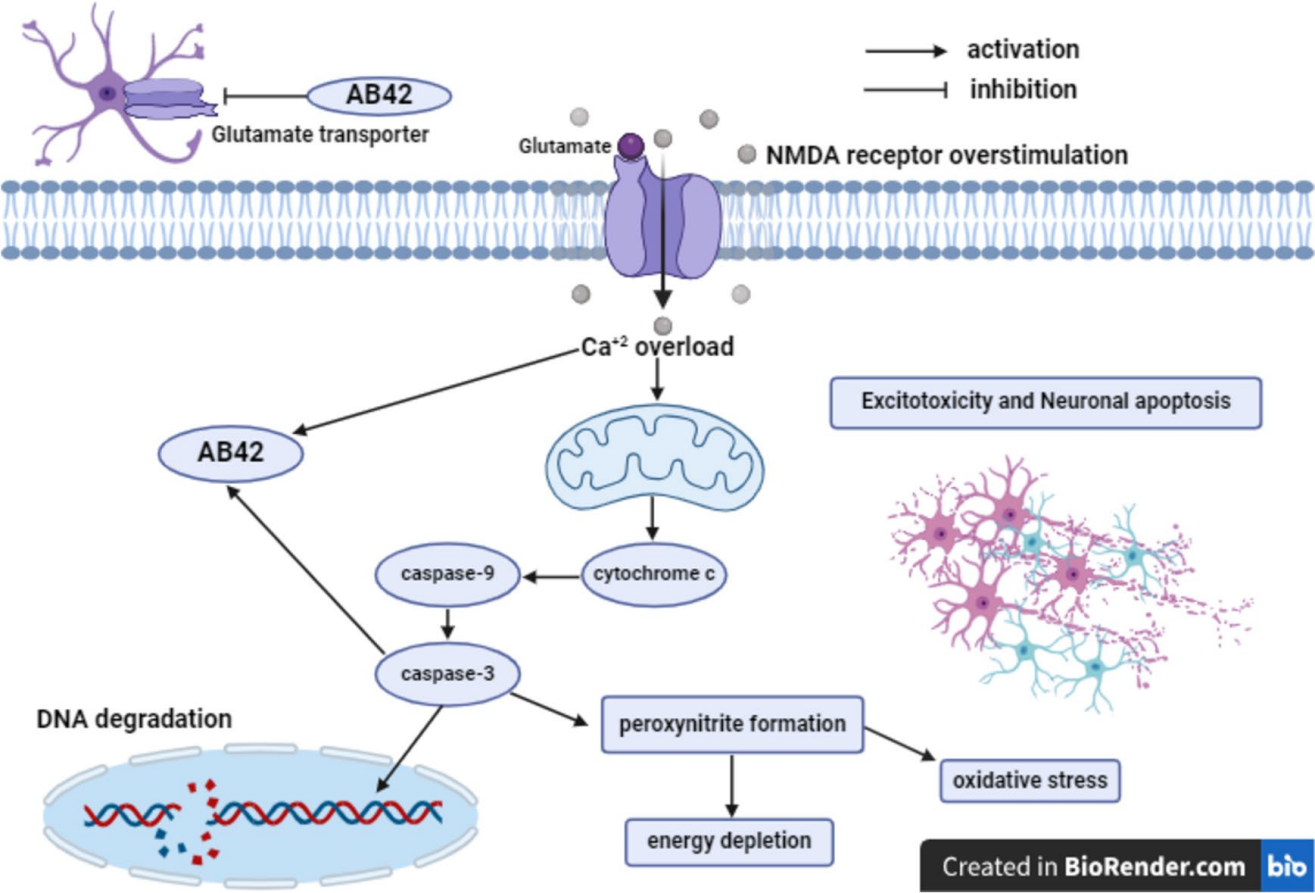
Glutamate and Excitotoxicity. A*β*42 amyloid-*β*42, NMDA N-methyl-D-aspartate

Glutamate, the prime excitatory neurotransmitter in the central nervous system (CNS), involves in learning and memory process (Fouad et al. 2018). The accumulation of A*β*42 peptides exacerbates astrocytic depolarization with consequent suppression of glutamate uptake, provoking incline in the extracellular glutamate level and excitotoxicity (Ekladious and El Sayed 2019). A remarkable decrement in glutamate reuptake sites and transporters such as glutamate transporter-1 (GLT-1) was demonstrated in the brains of AD patients (Butterfield and Pocernich 2003). Extracellular glutamate accumulation aggravates NMDA receptor overstimulation that provokes Ca^+2^-dependent apoptosis through cytochrome c release, inducing the proteolytic cleavage of caspase-9 with concomitant activation of its downstream effectors caspase-3, -6, and -7. Caspase-3 activation exacerbates DNA degradation and ONOO^−^ formation with consequent oxidative stress and energy depletion. Moreover, NMDA receptor overstimulation is implicated in A*β*42 plaques formation through Ca^+2^-mediated amyloidogenic processing of the APP, proteolytic cleavage of APP by caspase-3, as well as inhibition of A*β*42 clearance machinery from the brain (Fouad et al. 2018) (Fig. 6).

### BDNF and insulin signaling

BDNF, the predominant neurotrophin in the hippocampus, facilitates synaptic plasticity, neurogenesis, and survival (Diniz and Teixeira 2011). It binds to tyrosine kinase *β* (Trk*β*) receptor, resulting in activation of phosphoinositide 3-kinase (PI3K)/protein kinase B (AKT)/cAMP response element binding protein (CREB), Raf/mitogen-activated protein kinase (MEK)/extracellular signal-regulated kinase1/2 (ERK1/2)/CREB, and phospholipase-C*ϒ*/inositol 1,4,5-trisphosphate/Ca^+2^/calmodulin/calcium-calmodulin-dependent protein kinase/CREB survival pathways (Jin et al. 2019). CREB plays a pivotal role in learning and memory process as well as enhancing BDNF expression (Bitner 2012).

**Fig. 7:**
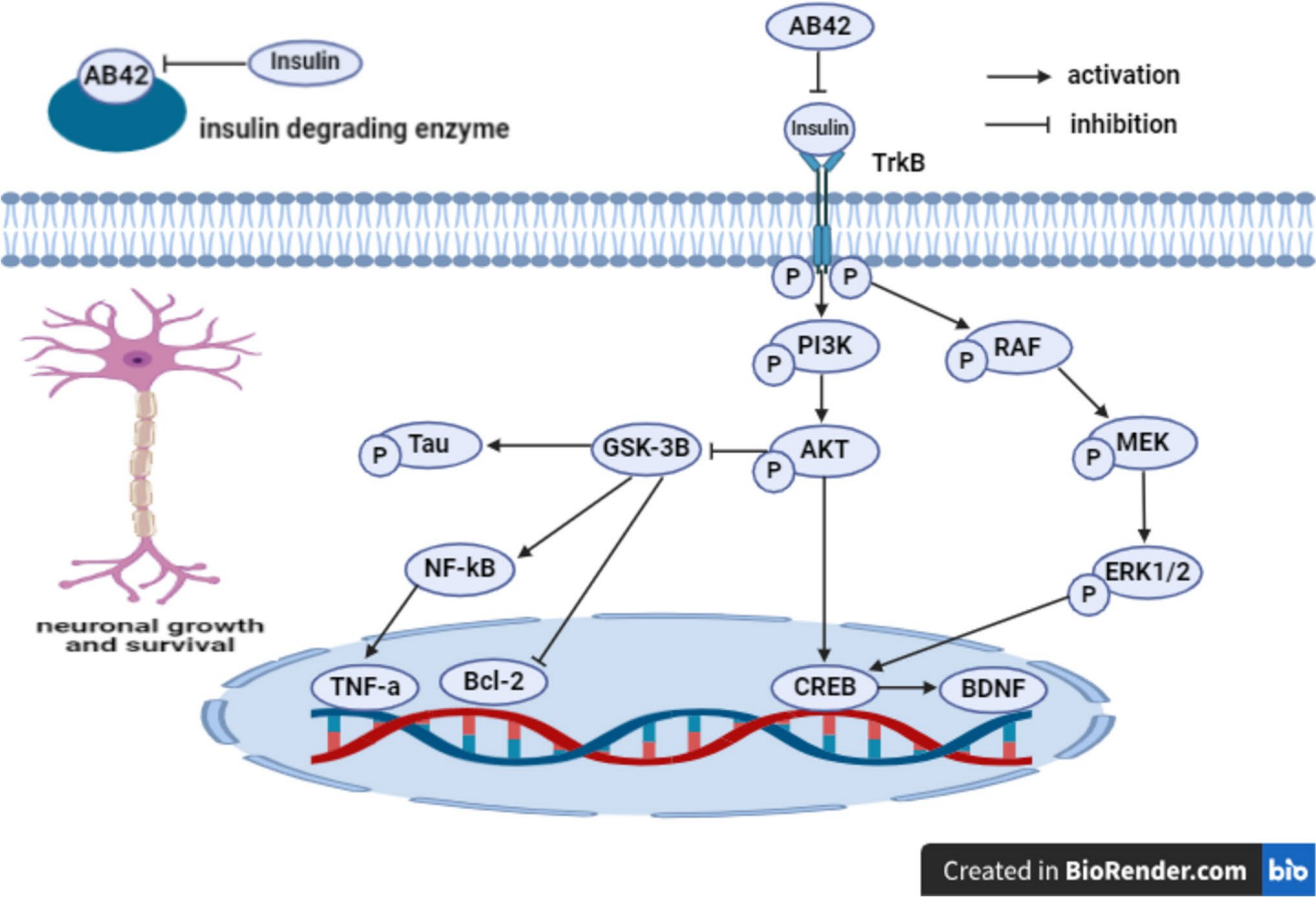
Insulin and GSK-3*β* signaling. A*β*42 amyloid-*β*42, Trk*β*, tyrosine kinase-*β*, p-PI3K phosphorylated phosphoinositide 3-kinase, p-AKT phosphorylated protein kinase B, CREB cAMP response element binding protein, p-MEK phosphorylated mitogen-activated protein kinase, p-ERK1/2 phosphorylated extracellular signal-regulated kinase1/2, GSK-3*β* glycogen synthase kinase-3 beta, p-Tau phosphorylated Tau, NF-κ*β* nuclear factor-kappa *β*, TNF-α tumor necrosis factor-α, Bcl-2 B cell lymphoma-2

Insulin promotes neuronal growth and memory processing through binding to Trk*β* receptor in the hippocampus and cortex with subsequent enhancement of PI3K/AKT/CREB and Raf/MEK/ERK1/2/CREB survival pathways (Plum et al. 2005; Freude et al. 2009; Parihar and Brewer 2010). It has been illustrated that metabolic syndrome, insulin resistance, and hyperinsulinemia induce A*β*42 plaques and NFTs formation in AD (Razay et al. 2007; Gabbouj et al. 2019). A*β*42 peptides compete with insulin for binding to insulin receptors, inducing insulin resistance and downregulation of insulin receptors resulting in A*β*42 formation and declined IDEs production, inhibiting A*β*42 degradation, additionally, insulin competes with A*β*42 for binding to IDEs receptors, causing reduction in IDEs availability to degrade A*β*42 peptides (Xie et al. 2002; Zhao et al. 2004; Luchsinger 2008). Furthermore, the inhibition of insulin signaling by A*β*42 plaques and NFTs hampers PI3K/AKT pathway, inducing GSK-3*β* activation (Kitagishi et al. 2014, Cai et al. 2015). GSK-3*β* is implicated in AD pathology via exacerbating Tau hyper-phosphorylation (Folch et al. 2016). Additionally, it activates microglia-mediated release of inflammatory cytokines nearby A*β*42 plaques through upregulation of NF-κ*β* and TNF-α (Woodgett and Ohashi 2005; Najem et al. 2014). Moreover, GSK-3*β* aggravates neuronal apoptosis in AD by downregulating Bcl-2 expression (Turenne and Price 2001; Sayas and Ávila 2021) **(**Fig. 7**)**.

### Wingless-related integration site (Wnt)/ *β*-catenin signaling

**Fig. 8:**
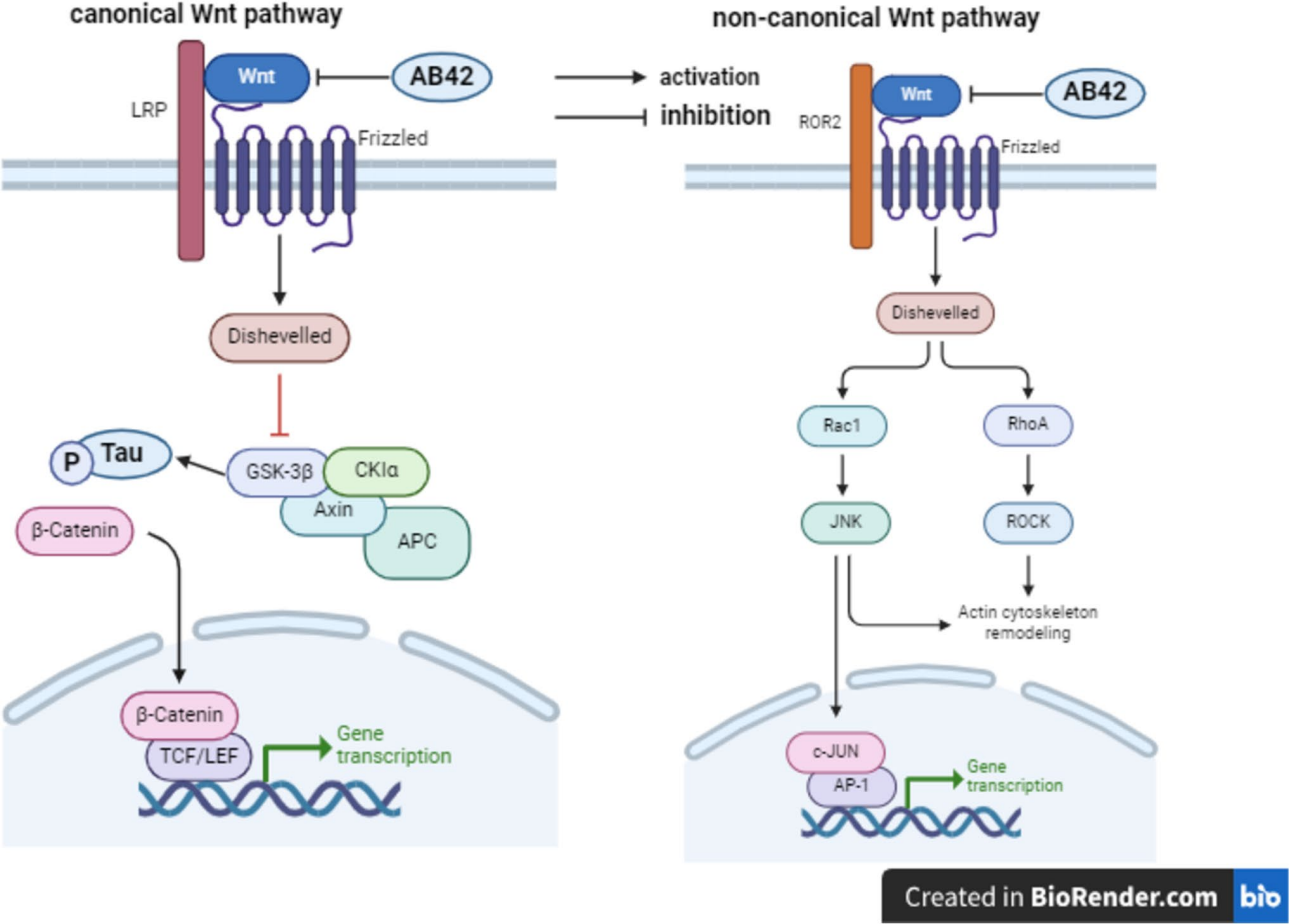
Canonical and Non-canonical Wnt signaling. A*β*42 amyloid-*β*42, Wnt wingless-related integration site, LRP low-density lipoprotein receptor-related protein, ROR2 receptor tyrosine kinase-like orphan receptor 2, GSK-3*β* glycogen synthase kinase-3 beta, CK1α casein kinase-1α, APC adenomatous polyposis coli, TCF T-cell factor, LEF lymphoid enhancer factor, Rac rac family small GTPase 1, RhoA homolog family member A, ROCK Rho-associated protein kinase, JNK c-Jun N-terminal kinase, c-JUN c-jun N-terminal kinase, AP-1 activator protein, p-Tau phosphorylated Tau

The canonical and non-canonical Wnt pathways facilitate memory, learning, as well as synaptic plasticity and neuronal survival (Folke et al. 2019; Jia et al. 2019; Ng et al. 2019). A*β*42 plaques inhibit Wnt/*β*-catenin pathway by stimulating Dickkopf-1, leading to cytosolic *β*-catenin degradation through phosphorylation at (S37) by GSK-3*β* and prevention of *β*-catenin translocation to the nucleus with consequent inhibition in Wnt target genes expression (Villar et al. 2011; Jia et al. 2019; Chen et al. 2020). Additionally, insulin resistance was reported to inhibit Wnt signaling and induce *β*-catenin degradation by GSK-3*β* (Kim et al. 2013). The canonical pathway is activated, when Wnt1,Wnt3a,Wnt7a/b or Wnt8 ligand binds to frizzled receptor thus inactivating a multiple protein complex that consists of casein kinase-1α, GSK-3*β*, Axin, and adenomatous polyposis coli, while facilitating the phosphorylation of *β*-catenin at (S675) and potentiating *β*-catenin accumulation in the cytoplasm and its translocation to the nucleus, resulting in Wnt target genes expression such as c-MYC, cyclin D1, Axin2, as well as Ca^+2^-calmodulin-dependent protein kinase type IV, and BDNF (Oliva et al. 2013; Muneeb et al. 2022). In the non-canonical pathway Wnt4,Wnt5a, or Wnt11 activates homolog family member A (RhoA) and rac family small GTPase1 (Rac1) with a concomitant activation of Rho-associated protein kinase (ROCK) and JNK, respectively, enhancing post-synaptic proteins clustering and gene transcription (Oliva et al. 2013) (Fig. 8).

### Autophagy

**Fig. 9:**
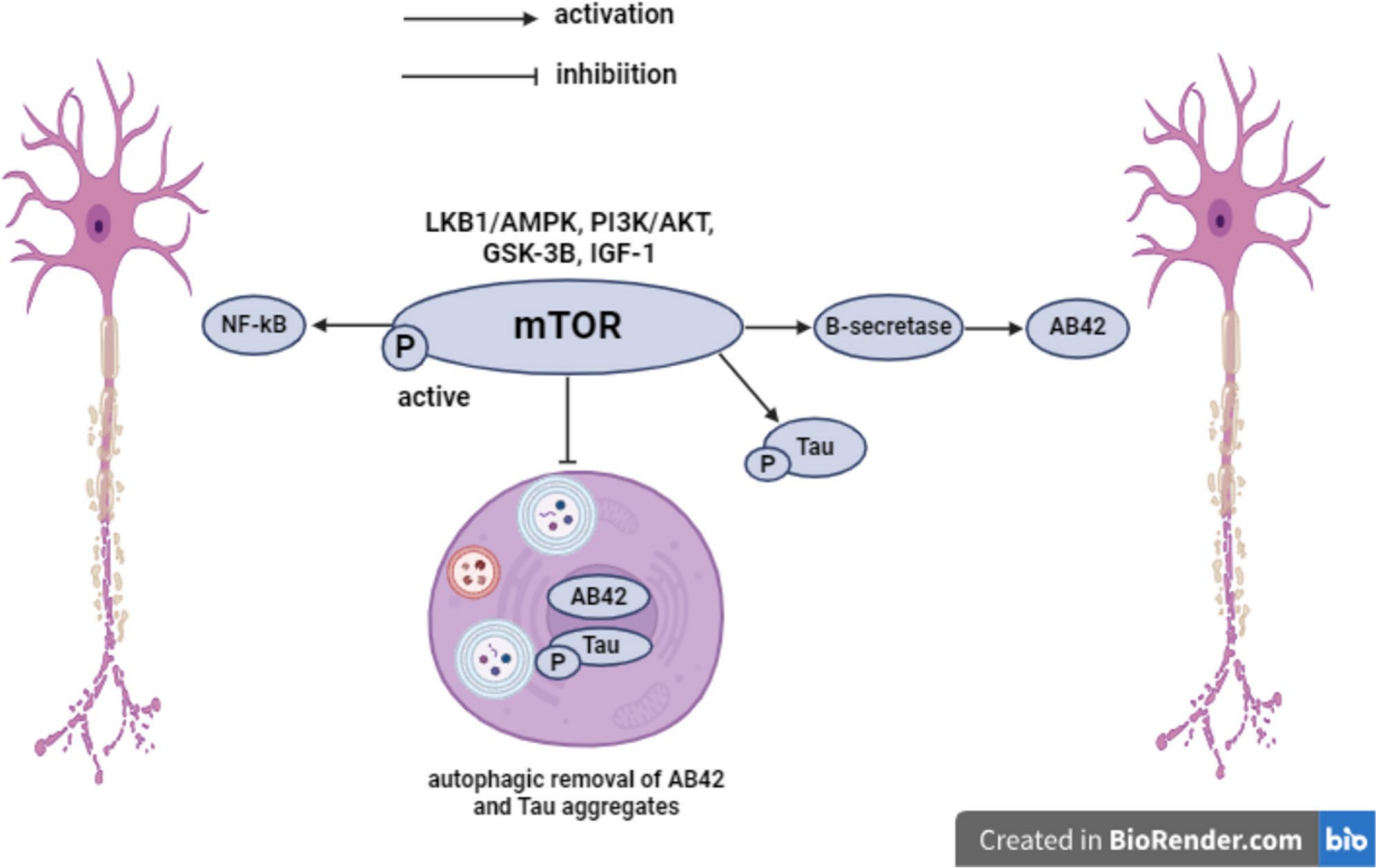
Autophagy and mTOR signaling. mTOR mammalian target of rapamycin, LKB1 Liver kinase B1, AMPK adenosine monophosphate-activated protein kinase, PI3K phosphoinositide 3-kinase, AKT protein kinase B, GSK-3*β* glycogen synthase kinase-3 beta, IGF-1 insulin like growth factor-1, A*β*42 amyloid-*β*42, p-Tau phosphorylated Tau

Autophagy, a natural degradation machinery, is conducted by lysosome to clear the damaged organelles as well as dysfunctional components including aggregated protein species such as A*β*42 and Tau aggregates (Kelekar 2006; Querfurth and Lee 2021). An impairment in the A*β*42 peptide clearance process through microglia via autophagy was demonstrated in patients suffering from AD (Estfanous et al. 2021). The autophagic machinery is mainly controlled by adenosine monophosphate-activated protein kinase (AMPK) and mTOR (Kim et al. 2011). The AMPK activation upregulates autophagy inducing proteins including microtubule-associated protein light chain 3 (LC3B) and Beclin-1 to form autophagosome (Ibrahim et al. 2022). mTOR, serine/threonine protein kinase, regulates several physiological functions, including energy metabolism, protein synthesis, cellular growth, and autophagy. The activity of mTOR is controlled by various upstream signals, such as insulin growth factors1, Liver kinase B1 (LKB1)/AMPK, PI3K/AKT, and GSK-3*β*. The activation of mTOR induces A*β*42 generation through enhancement of *β*- and γ-secretases (Cai et al. 2015). It was documented that mTOR activation contributes to Tau hyper-phosphorylation as well as inhibiting the autophagic clearance of A*β*42 and p-Tau aggregates, provoking A*β*42 plaques and NFTs formation (Oddo 2012; Querfurth and Lee 2021). Moreover, mTOR aggravates neuroinflammation via activating NF-κ*β* (Xu et al. 2021) **(**Fig. 9**)**.

## Drugs repurposing in the experimental models of AD

### Anti-inflammatory drugs

#### COX isoforms in AD

COX-1 and COX-2 enzymes are responsible for prostaglandins (PGEs) formation, exacerbating neuroinflammation and synaptic loss in the CNS. The peroxidase activity of COX induces ROS production during PGEs formation process. Additionally, PGE1 and PGE2 aggravate glutamate release from astrocytes, inducing excitotoxicity. Valeryla salicylate, a selective COX-1 inhibitor, and Etoricoxib, a selective COX-2 inhibitor, could improve the cognitive function of streptozotocin (STZ)-injected rats. They inhibited STZ-induced AchE activation and oxidative stress. On the contrary, phenacetin, a selective COX-3 inhibitor, could not perform any neuroprotective activity (Dhull et al. 2012). In addition, salicylate was able to mitigate A*β*42-mediated cognitive and synaptic dysfunction in rats (Mohammadpour et al. 2015) along with its neuroprotective activity against Tau pathology in PS19 transgenic mice model of FTD (Min et al. 2015). Further, celecoxib, a selective COX-2 inhibitor, ameliorated the memory impairment and enhanced neurogenesis in aluminium chloride (AlCl3)-injected rats. It diminished AlCl3-provoked AchE activation, astrogliosis, and apoptosis (Abdel-Aal et al. 2021). Furthermore, celecoxib showed a neuroprotective activity against A*β*42 deposition, neuroinflammation and excitotoxicity in lipopolysaccharide (LPS)-injected mice (Sayed and El Sayed 2016).

#### Leukotriene antagonist

Cysteinyl leukotrienes (CysLTs) such as LTC-4, LTD-4, and LTE-4 are inflammatory mediators derived from 5-LOX pathway of arachidonic acid metabolism. Cysteinyl leukotriene receptor-1 (CysLT1R) and CysLT2R are the main studied receptors for CysLTs. LTD-4 acts on CysLT1R, resulting in *β*-secretase activation and neuroinflammation in the hippocampus and cerebral cortex (Hu et al. 2014). Montelukast, a CysLT1R antagonist for bronchial asthma, could mitigate the cognitive abnormality of STZ-injected mice. It alleviated STZ-induced neuroinflammation and apoptosis (Zhang et al. 2016). Additionally, montelukast ameliorated the memory decline and facilitated neuronal growth in Scopolamine (SPN)-injected rats. It reduced SPN-induced A*β*42 deposition, AchE activation, neuroinflammation, and oxidative damage (Yerraguravagari et al. 2024). Further, montelukast is in the phase two clinical trial NCT03402503 as disease-modifying drug (Cummings et al. 2024).

#### JAK inhibitor

Baricitinib, a JAK inhibitor for rheumatoid arthritis, was recorded to rescue the memory impairment of d-galactose (d-gal)/ovariectomy (OVX) rats. It demonstrated a neuroprotective activity against A*β*42 deposition, oxidative stress, neuroinflammation, and astrogliosis in d-gal/OVX rats by inhibiting JAK2/STAT3 and PI3K/AKT/mTOR signaling pathways (Hindam et al. 2024).

### Anti-hypertensive drugs

#### Angiotensin converting enzymes in AD

The central angiotensin-converting enzyme-1/angiotensin-2/angiotensin receptor-1(ACE-1/Ang-2/AT-1) is implicated in *β*-secretase activation, A*β*42 deposition, AchE enhancement, apoptosis, transcription of inflammatory cytokines and NOX/H2O2 activation that enhances chloride current, causing inhibition of ACE-2 enzyme. Angiotensin-converting enzyme-2/angiotensin-(1–7)/Masreceptor (ACE-2/Ang-(1–7)/ MasR) axis activation abolishes the destructive potential of AT-1 receptor signaling in AD by forming heterodimer with AT-1 receptor (Kamel et al. 2018; Messiha et al. 2020). Diminazene, an ACE-2 activator, could improve the cognitive function and facilitate neuronal growth in d-gal/OVX rats. It suppressed A*β*42 deposition, Tau aggregates formation, neuroinflammation, astrogliosis and apoptosis in d-gal/OVX rats through modulation of PI3K/AKT/GSK-3*β* and upregulation of nAChR and glutamate receptor-2 (GluR-2) (Kamel et al. 2018). Perindopril, an ACE1 inhibitor, ameliorated the memory impairment of D-gal/OVX rats. It diminished d-gal/OVX-exacerbated A*β*42 deposition and Tau aggregates formation, AchE activation, oxidative damage, and neuroinflammation by upregulating central estrogen receptor, NEP, and IDE (Messiha et al. 2020). Additionally, perindopril was illustrated to inhibit AchE activation and oxidative stress in AlCl3-injected mice (Yang et al. 2013). Telmisartan, an AT1 receptor blocker, was able to ameliorate the cognitive impairment of AlCl_3_-injected rats. It reduced AlCl3-evoked A*β*42 deposition, Tau aggregates formation, AchE activation, neuroinflammation, and oxidative damage (Khalifa et al. 2020). Further, telmisartan displayed anti-inflammatory activity in A*β*42-injected rats (Shindo et al. 2012). Furthermore, it is in the phase two clinical trials NCT02085265 as disease-modifying drug (Cummings et al. 2024). Moreover, candesartan, an AT1 receptor blocker, has been documented to alleviate STZ-induced AchE activation and oxidative stress in mice (Tota et al. 2009).

#### Ca^+2^ channel blocker

Diltiazem, a non-dihydropyridine benzothiazpine Ca^+2^ channel blocker, could reverse the cognitive dysfunction of STZ-injected rats. It mitigated STZ-induced A*β*42 deposition, neuroinflammation, and oxidative damage (Alluri et al. 2023). Additionally, diltiazem was reported to inhibit AchE activation and oxidative stress in AlCl3-injected mice (Rani et al. 2015).

#### Phosphodiesterase-5 (PDE-5) inhibitors

Sildenafil, a PDE-5 inhibitor for erectile dysfunction, improved memory impairment of APP/PS1 transgenic mice. It reduced A*β*42 deposition and neuroinflammation in APP/PS1 transgenic mice (Zhang et al. 2013). Additionally, sildenafil displayed anti-inflammatory and antioxidant effects in AlCl3-treated rats (Ibrahim et al. 2021). The PDE-5 inhibitor tadalafil, was also able to reverse cognitive abnormality and enhance neuronal growth in STZ-injected mice. It reduced A*β*42 deposition, Tau aggregates formation, and neuroinflammation in STZ-injected mice via cyclic guanosine monophosphate (cGMP)/protein kinase G (PKG)/AKT and Wnt3a signaling pathways activation as well as GSK3-*β* inhibition, AMPK/mTOR modulation, and enhancement of the autophagic machinery (Salem et al. 2021).

### Antidiabetic drugs

#### Glucagon-like peptide-1 (GLP-1) agonists

GLP-1 is an endogenous peptide predominantly secreted from L cells in gastrointestinal after food ingestion to promote insulin secretion, additionally, it exerts a neuroprotective activity in the CNS (El-Sahar et al. 2021). GLP-1 receptor activation in the hippocampus enhances neuronal growth and synaptic transmission as well as reducing A*β*42 deposition, neuroinflammation, and apoptosis (McClean and Hölscher 2014; Yossef et al. 2020; El-Sahar et al. 2021). Liraglutide, a GLP-1 receptor agonist for type 2 diabetes mellitus (T2DM), ameliorated the cognitive decline of STZ-injected mice. It alleviated STZ-mediated A*β*42 deposition, astrogliosis, and neuroinflammation (Paladugu et al. 2021). Additionally, liraglutide was recorded to decrease A*β*42 deposition and enhanced synaptic transmission in APP/PS1 transgenic mice (McClean and Hölscher 2014). Further, semaglutide, a GLP-1 receptor agonist for T2DM, is in the phase three clinical trial NCT04777396 as disease-modifying drug (Cummings et al. 2024).

#### Dipeptidyl peptidase-4 (DPP-4) inhibitors

Vildagliptin, a DPP-4 inhibitor for T2DM reduces GLP-1 degradation to increase its lifespan to act on GLP-1 receptor, could improve the cognitive abnormality of STZ-injected rats. It lowered STZ-provoked A*β*42 deposition, Tau aggregates formation, and neuroinflammation (Kosaraju et al. 2013). Additionally, vildagliptin diminished A*β*42 deposition, neuroinflammation, and apoptosis in AlCl3-injected rats with high fat/sugar diet through cAMP/protein kinase A (PKA) and MAPK/ERK activation as well as PI3K/AKT/GSK-3*β* modulation and JAK2/STAT3 inhibition (Yossef et al. 2020). The DPP-4 inhibitor alogliptin has also been reported to reduce LPS-induced A*β*42 deposition, neuroinflammation, astrogliosis, and apoptosis in mice (El-Sahar et al. 2021).

#### Insulin sensitizer

Metformin, an insulin sensitizer for T2DM, was able to alleviate memory impairment and enhance synaptic plasticity in STZ-injected rats. It ameliorated STZ-evoked AchE activation, astrogliosis, neuroinflammation, and apoptosis (Mohamed et al. 2020; Pilipenko et al. 2020). Additionally, metformin is in the phase three clinical trial NCT04098666 as disease-modifying drug (Cummings et al. 2024).

#### Peroxisome proliferator-activated receptor-γ (PPAR-γ) agonist

The activation of central PPAR-γ was documented to reduce A*β*42 deposition, neuroinflammation, and glutamate-mediated excitotoxicity through enhancement of GLT-1 expression, causing glutamate uptake with subsequent decline in glutamate concentration and excitotoxicity (Jiang et al. 2008; Ekladious and El Sayed 2019). Pioglitazone, a PPAR-γ receptor agonist for T2DM, could mitigate the cognitive dysfunction of LPS injected-mice. It suppressed A*β*42 deposition, oxidative damage, and excitotoxicity in LPS-injected mice (Ekladious and El Sayed 2019). Additionally, pioglitazone was able to decrease STZ-induced AchE activation and oxidative stress in mice (Kaur et al. 2009).

#### Sodium-glucose cotransporter-2 (SGLT-2) inhibitors

The inhibition of SGLT-2 in the CNS particularly in the hippocampus and blood brain barrier exhibited a neuroprotective activity in neurodegenerative diseases such as AD, Parkinson’s disease and Huntington’s disease (Ibrahim et al. 2022). Canagliflozin, a SGLT-2 inhibitor for T2DM, ameliorated the cognitive disturbance and facilitated neuronal growth in STZ-injected mice. It diminished STZ-mediated A*β*42 deposition, Tau aggregates formation, AchE activation, neuroinflammation, and oxidative stress. The protective activity of canagliflozin in STZ-injected mice was attributed to activation of AMPK/SIRT1 and IDEs, as well as inhibition of *β*-secretase and GSK-3*β* (Khamies et al. 2024). Dapagliflozin, a SGLT-2 inhibitor for T2DM, was documented to improve memory process of D-gal/OVX rats and reduce D-gal/OVX-induced A*β*42 deposition and Tau aggregates formation via LKB1/AMPK/SIRT1 activation, mTOR inhibition, and autophagy induction (Ibrahim et al. 2022). Additionally, it decreased oxidative damage, neuroinflammation, and apoptosis in LPS-injected rats (Abd Elmaaboud et al. 2023). Further, dapagliflozin mitigated AlCl3-induced A*β*42 deposition, AchE activation, and oxidative stress in rats through AMPK/mTOR modulation (Samman et al. 2023). Furthermore, insulin and empagliflozin, SGLT-2 inhibitor for T2DM, are in the phase two clinical trial NCT05081219 as disease-modifying therapy (Cummings et al. 2024).

### Antiepileptic drugs

Histone Deacetylase (HDAC) inhibitors drugs facilitate recognition and spatial memory by diminishing *β*-secretase cleavage, A*β*42 deposition, Tau hyper-phosphorylation, and microglial activation, while enhancing *α*-secretase cleavage, BDNF, SIRT1, and NEP gene expression (Belyaev et al. 2009; Esposito and Sherr 2019). Valproic acid, a Na^+^ channel blocker antiepileptic drug and HDAC inhibitor, ameliorated the cognitive dysfunction of STZ-injected mice. It mitigated STZ-induced A*β*42 deposition and AchE activation (Sorial and El Sayed 2017). Additionally, valproic acid was able to decrease A*β*42 deposition, neuroinflammation, astrogliosis, and apoptosis in APP/PS1 transgenic mice by GSK-3*β* inhibition (Long et al. 2013; Xuan et al. 2015). Levetiracetam, an atypical antiepileptic drug, improved the cognitive decline of STZ-injected rats. It inhibited STZ-mediated Tau aggregates formation, AchE activation, neuroinflammation, and oxidative damage (Alavi et al. 2022). Additionally, levetiracetam was recorded to reverse memory impairment and promote synaptic plasticity in human amyloid precursor protein (hAPP) transgenic mice (Sanchez et al. 2012). Moreover, levetiracetam is in the phase three clinical trial NCT05986721 as disease-modifying drug (Cummings et al. 2024). Topiramate, an atypical antiepileptic drug, counteracted the cognitive abnormality of cadmium injected-rats. It alleviated cadmium-induced A*β*42 deposition, Tau aggregates formation, AchE activation, oxidative damage, and apoptosis through GSK-3*β* inhibition, AMPK/mTOR modulation, and autophagy promotion (Arab et al. 2023). Additionally, topiramate was able to decrease A*β*42 deposition in APP/PS1 transgenic mice (Owona et al. 2019). Moreover, it was documented to diminish STZ-evoked cognitive impairment and oxidative stress in mice (Price et al. 2015).

### Antidepressant drugs

#### Selective serotonin reuptake inhibitors (SSRIs)

Serotonin (5-hydroxytryptamine, 5-HT) plays a pivotal role in sleep, appetite, sexual function, as well as memory (Ramirez et al. 2014). Decline in serotonergic neurons and 5-HT concentration was reported in the dorsal and median raphe nuclei nearby A*β*42 plaques in AD patients (Chen et al. 2000; Lyness et al. 2003). Additionally, the cortical concentration of 5-HT is negatively correlated with Tau protein concentration (Palmer et al. 1987), moreover, downregulation of 5-HT receptors was noticed in AD patients (Rodríguez et al. 2012). Escitalopram, a SSRI antidepressant drug, could mitigate the cognitive dysfunction and facilitate neuronal growth in D-gal/OVX rats. It diminished d-gal/OVX-exacerbated A*β*42 deposition, Tau aggregates formation, neuroinflammation, and oxidative stress through PI3K/AKT/GSK-3*β* modulation, JNK inhibition, and Raf/MEK/ERK1/2 activation (Ibrahim et al. 2019). Additionally, escitalopram reversed the memory impairment and AchE activation in SPN-injected rats (Krishna et al. 2021). Further, escitalopram is in the phase three clinical trial NCT03108846 for neuropsychiatric symptoms (Cummings et al. 2024). Fluoxetine, a SSRI antidepressant drug, has been demonstrated to suppress A*β*42 deposition and apoptosis in APP/PS1 transgenic mice by enhancing Wnt/*β*-catenin signaling as well as inhibiting GSK-3*β* and *β*-secretase (Huang et al. 2018, Zhou et al. 2019).

#### Tricyclic antidepressant drugs

Imipramine, a tricyclic antidepressant drug, ameliorated cognitive decline and enhanced neuronal growth in STZ-injected rats. It decreased STZ-provoked neuroinflammation and apoptosis via enhancing insulin signaling and inhibiting JNK (Javadpour et al. 2021; Hasanabadi et al. 2024). Additionally, amitriptyline, a tricyclic antidepressant drug was able to diminish A*β*42 deposition and promote neurogenesis in APP/PS1 transgenic mice (Chadwick et al. 2011).

### Anticancer drugs

Epidermal growth factor receptor (EGFR), Trk, plays a pivotal role in memory formation, neuronal growth, and synaptic plasticity through modulation of glutamate release. However, EGFR overstimulation is implicated in AD through exacerbation of A*β*42 deposition, neuroinflammation, and NOX activation as well as inhibiting the autophagic clearance of A*β*42 and p-Tau aggregates. Human epidermal growth factor receptor (HER-2) was detected in postmortem AD brain as it is involved in A*β*42 deposition and autophagy inhibition. Lapatinib, a dual EGFR and HER-2 inhibitors for metastatic breast cancer, could rescue the cognitive decline of D-gal/OVX rats. It suppressed D-gal/OVX-provoked A*β*42 deposition, Tau aggregates formation, neuroinflammation, oxidative damage, and excitotoxicity through modulation of PI3K/AKT/GSK-3*β* and inhibition of mTOR and P38-MAPK signaling as well as induction of the autophagic machinery (Mansour et al. 2021a, b; Mansour et al. 2021a, b). Additionally, there are several anti-cancer drugs demonstrated ability to ameliorate the memory impairment in the experimental studies such as nilotinib, neratinib, axitinib, binimetinib, sunitinib, ibrutinib, abemaciclib, and regorafenib (Lee et al. 2024). Further, nilotinib (NCT05143528) and masitinib (NCT05564169) are in the phase three clinical trial as disease-modifying drugs. Furthermore, dasatinib (NCT04685590) is in the phase two clinical trial as disease-modifying drug (Cummings et al. 2024) (Table 1).

**Table 1:** Pipeline drugs

Drug	Class	Code	Phase	Lead sponsor	Therapeutic purpose	Date
Montelukast	Anti-inflammatory	NCT03402503	Two	IntelGenx Corp	Disease-modifying drug	From Nov 2018 to Mar 2024
Telmisartan	Antihypertensive	NCT02085265	Two	Sunnybrook Health sciences Center	Disease-modifying drug	From Mar 2014 to Sep 2023
Metformin	Antidiabetic	NCT04098666	Three	Columbia University	Disease-modifying drug	From Mar 2021 to Apr 2026
Semaglutide	Antidiabetic	NCT04777396	Three	Novo Nordisk A/S	Disease-modifying drug	From May 2021 to Sep 2025
Empagliflozin and insulin	Antidiabetic	NCT05081219	Two	Wake Forest University Health Sciences	Disease-modifying drug	From Oct 2021 to Oct 2026
Levetiracetam	Antiepileptic	NCT05986721	Three	AgeneBio	Disease-modifying drug	From Dec 2024 to Jul 2028
Escitalopram	Antidepressant	NCT03108846	Three	JHSPH Center for Clinical Trials	Neuropsychiatric symptoms	From Jan 2018 to May 2025
Nilotinib	Anticancer	NCT05143528	Three	KeifeRx, LLC	Disease-modifying drug	From Feb 2022 to Dec 2025
Masitinib	Anticancer	NCT05564169	Three	AB Science	Disease-modifying drug	From Jan 2024 to Dec 2026
Dasatinib	Anticancer	NCT04685590	Two	Wake Forest University	Disease-modifying drug	From Dec 2021 to Jan 2025

## The pipeline and FDA approved drugs for AD

The FDA approved drugs are donepezil, rivastigmine, and galantamine, AchEs inhibitors, for symptomatic treatment of mild to moderate cases, while memantine, a NMDA receptor antagonist by allosteric modulation, for moderate to severe cases. Further, memantine can be used alone or in combination with AchEs inhibitors (Alhazmi and Albratty 2022). Moreover, the FDA approved brexipiprazole, an atypical antipsychotic for agitation, and suvorexant, an orexin receptor antagonist for insomnia associated with the AD symptoms (Varadharajan et al. 2023; Cummings et al. 2024). Unfortunately, these drugs were not able to halt the neurodegeneration, requiring further investigation to develop other drugs (Alhazmi and Albratty 2022). The technique of passive immunity was used to develop monoclonal antibodies (mAbs) against A*β*42 peptides, Tau protein, and inflammatory cytokines as immunotherapy (Cummings et al. 2024). The FDA has recently approved aducanumab in June 2021, lecanemab in July 2023, and donanemab in July 2024 against A*β*42 peptides (Cummings et al. 2024; Kang 2024). Further, gantenerumab (NCT01760005) and solanezumab (NCT01760005) against A*β*42 peptides are in the phase three clinical trial as disease-modifying drugs. Furthermore, bepranemab (NCT04867616) against Tau protein and canakinumab (NCT04795466) against IL-1*β* are in the phase two clinical trial as disease-modifying drugs (Cummings et al. 2024). Aducanumab was reported to slow the rate of cognitive decline without completely preventing the memory loss in AD patients, additionally, gantenerumab and solanezumab could reduce A*β*42 concentrations without achieving a significant recovery from cognitive dysfunction in AD patients, confirming that A*β*42 peptide is not the whole story to develop treatment for AD (Lee et al. 2024) (Table 2).

**Table 2:** Pipeline mAbs

Mab	Target	Code	Phase	Lead sponsor	Therapeutic purpose	Date
Gantenerumab	A*β*42	NCT01760005	three	Washington University School of Medicine	Disease-modifying drug	From Dec 2012 to Apr 2028
Solanezumab	A*β*42	NCT01760005	three	Washington University School of Medicine	Disease-modifying drug	From Dec 2012 to Apr 2028
Bepranemab	Tau protein	NCT04867616	two	UCB Biopharma SRL	Disease-modifying drug	From Jun 2021 to May 2024
Canakinumab	IL-1*β*	NCT04795466	two	Novartis Pharmaceuticals	Disease-modifying drug	From Oct 2021 to Mar 2024

## Conclusions

The concept of drug development in AD should be based on multi-target strategy such as A*β*42 deposition, Tau hyper-phosphorylation, oxidative stress as well as neuroinflammation, and insulin signaling impairment to achieve a marked amelioration in the memory process and prevent neurodegeneration. The immunotherapy needs further investigation for safety profile so drugs repurposing technique is considered as better option for elucidating safe and effective treatment (Lee et al. 2024). Additionally, we reported in this review that anti-inflammatory, anti-hypertensive, anti-diabetic, antiepileptic, antidepressant and anticancer drugs have a promising therapeutic activity against AD, confirming the value of the drugs repurposing technique. Further, we illustrated the pathophysiology of AD to help the new researchers to discover treatment as until this moment there is no curative therapy for AD.

## Data Availability

Not applicable.

## Abbreviations

AD: Alzheimer’s disease
APP: Amyloid precursor protein
A*β*42: Amyloid-*β*42
Ach: Acetylcholine
AchE: Acetylcholine esterase
AMPK: Adenosine monophosphate-activated protein kinase
ACE: Angiotensin-converting enzyme
Ang: Angiotensin
AT: Angiotensin receptor
α7nAChR: α7 Nicotinic acetylcholine receptor
APOE: Apolipoprotein E
AlCl3: Aluminium chloride
Bcl-2: B cell lymphoma-2
Bax: Bcl-2-associated x protein
BDNF: Brain derived neurotrophic factor
CNS: Central nervous system
ChAT: Choline acetyl-transferase
cAMP: Cyclic adenosine monophosphate
CREB: CAMP response element binding protein
cGMP: Cyclic guanosine monophosphate
COX: Cyclooxygenase
CysLTs: Cysteinyl leukotrienes
CysLT1R: Cysteinyl leukotriene receptor-1
D-gal: d-galactose
DPP-4: Dipeptidyl peptidase-4
EGFR: Epidermal growth factor receptor
ERK1/2: Extracellular signal-regulated kinase ½
FTD: Frontotemporal dementia
GFAP: Glial fibrillary acidic protein
GLP-1: Glucagon-like peptide-1
GLT-1: Glutamate transporter-1
GLUR-2: Glutamate receptor
GSH: Glutathione
GPx: Glutathione peroxidase
GST: Glutathione S-transferase
GSK-3*β*: Glycogen synthase kinase-3 beta
HO-1: Heme oxygenase-1
HDAC: Histone deacetylase
hAPP: Human amyloid precursor protein
H_2_O_2_: Hydrogen peroxide
^•^OH: Hydroxyl radicals
5-HT: 5-Hydroxytryptamine
HER-2: Human epidermal growth factor receptor
iNOS: Inducible nitric oxide synthase
IDE: Insulin degrading enzyme
IL-1*β*: Interleukin-1*β*
IL-6: Interleukin-6
JAK2/STAT3: Janus Kinase-2/Signal Transducers and Activators of Transcription-3
JNK: C-Jun N-terminal kinase
Keap-1: Kelch-like ECH-associated protein-1
LPS: Lipopolysaccharide
5-LOX: 5-Lipoxygenase
LKB1: Liver kinase B1
LC3B: Microtubule-associated protein light chain 3
mTOR: Mammalian target of rapamycin
MasR: Masreceptor
MMP: Matrixmetalloproteinase
MEK: Mitogen-activated protein kinase
mAbs: Monoclonal antibodies
NEP: Neprilysin
NADPH: Nicotinamide adenine dinucleotide phosphate
NOX: NADPH oxidase
NFTs: Neurofibrillary tangles
NO: Nitric oxide
NMDA: *N*-methyl-d-aspartate
Nrf2: Nuclear factor erythroid 2-related factor 2
NF-κ*β*: Nuclear factor-kappa *β*
NLRP3: Nucleotide-binding oligomerization domain (NOD)-like receptor protein 3
OVX: Ovariectomy
PPAR-γ: Peroxisome proliferator-activated receptor-γ
ONOO^−^: Peroxynitrite
PDE: Phosphodiesterase
PI3K: Phosphoinositide 3-kinase
p-Tau: Phosphorylated Tau
P38-MAPK: P38-mitogen-activated protein kinase
PS: Presenilin
PGEs: Prostaglandins
PKA: Protein kinase A
AKT: Protein kinase B
PKG: Protein kinase G
PP2A: Protein phosphatase-2A
ROS: Reactive oxygen species
Rac: Rac family small GTPase 1
RhoA: Homolog family member A
ROCK: Rho-associated protein kinase
SPN: Scopolamine
SSRI: Selective serotonin reuptake inhibitor
SIRT1: Silent information regulator1
SGLT-2: Sodium-glucose cotransporter-2
STZ: Streptozotocin
SOD: Superoxide dismutase
TNF-α: Tumor necrosis factor-α
P53: Tumor suppressor protein
T2DM: Type 2 diabetes mellitus
Trk: Tyrosine kinase
Wnt: Wingless-related integration site
